# Training Addiction Psychiatry Fellows in Insomnia: Survey Results and Recommendations

**DOI:** 10.1007/s40596-025-02282-9

**Published:** 2025-12-01

**Authors:** Zoë Panchal, Alexis Ritvo, Susan K. Mikulich-Gilbertson, Jessica Camacho, Joseph T. Sakai

**Affiliations:** University of Colorado Anschutz Medical Campus, Aurora, CO USA

To the Editor:

Insomnia treatment is an essential part of the clinical care of patients with substance use disorders (SUDs), as insomnia is highly co-morbid with SUDs and may predict worse clinical outcomes [1]. Indeed, up to 70% of individuals with alcohol use disorder meet clinical criteria for insomnia disorder [1]. However, the diagnosis and management of insomnia are complicated in SUD populations for several reasons. First, substance intoxication and withdrawal may lead to insomnia symptoms (e.g., substance-induced insomnia) [1], which can make the cause of sleep problems more challenging to disentangle in individuals with SUDs. Second, there are unique considerations for insomnia management in the context of SUDs. For example, prescribing non-benzodiazepine receptor agonists and benzodiazepines with certain substances of abuse, such as opioids, can cause overdose [2]. In addition, medications used to treat SUDs, such as methadone, can themselves cause sleep problems [1]. Third, medications prescribed for insomnia in the general population may have unique, and clinically important, effects in SUD populations. For example, mirtazapine has been shown to reduce methamphetamine use in certain populations [3]. Thus, skills in SUD-specific insomnia diagnosis and management are essential for addiction psychiatrists. In this context, we sought to understand the current state of clinical insomnia training and practice in addiction psychiatry fellowships and identify areas for improvement. We therefore surveyed fellows and core faculty members at addiction psychiatry fellowships in the USA about their clinical training in, and management of, insomnia and other sleep disorders.

Participants were recruited via email from the listserv for addiction psychiatry fellowship program directors and administrators, which includes all the Accreditation Council for Graduate Medical Education (ACGME)–accredited addiction psychiatry programs. Listserv members were asked to forward the recruitment email to addiction psychiatry faculty and fellows at their programs; thus, the survey was forwarded to an unknown number of final recipients. Two reminder emails were sent out over the study period of 3 months. Individuals were eligible for the study if they were at least 18 years old and a fellow or core faculty member at an ACGME-accredited addiction psychiatry fellowship training program and provided direct clinical care to patients with SUDs. The study consisted of a single anonymous survey, delivered electronically between February and April of 2024. Participants were compensated with a $15.00 gift card. The study was exempted from IRB oversight by the Colorado Multiple Institutional Review Board. The survey consisted of 32 items which asked about: respondent demographics, formal sleep medicine training, perceived importance of treating insomnia in patients with SUDs, frequency of encountering insomnia in patients with SUDs, and management practices for insomnia in patients with SUDs. Questions on frequency of encountering insomnia clinically and on sleep disorders management practices referred to the past 6 months. Response types for questions included yes/no, count, and Likert scale. For questions using Likert scales, response options ranged from 0 (not important/never) to 4 (essential/always). As distributions for multiple variables were non-normal, we report median and inter-quartile range (IQR) as measures of central tendency.

Fifty-three physicians from 23 addiction psychiatry fellowship programs responded and met inclusion criteria. Of these, 50 completed the survey. Respondents had a median age of 41 (IQR = 18) years and were 66% male and 66% white. Sixty percent of respondents were faculty and 40% fellows. Respondents reported a median of 27.5 (IQR = 28) hours per week of clinical care of patients with SUDs and 3.5 (IQR = 14.6) years practicing addiction psychiatry.

Seventy percent of respondents reported receiving some form of sleep medicine training during their addiction psychiatry fellowship. Respondents reported that a median 70% of their patients in the past 6 months endorsed insomnia. However, only 32% of respondents received training in cognitive-behavioral therapy for insomnia (CBT-I), the first-line treatment for insomnia [4]. Respondents reported recommending or prescribing insomnia treatment to only 70% of their SUD patients with insomnia and recommending CBT-I only *sometimes* to those patients. Finally, many reported treating insomnia in patients with SUDs using medications that are relatively contraindicated in this setting because of the risks of overdose and dependence including non-benzodiazepine receptor agonists (*n* = 26), benzodiazepines (*n* = 15), and barbiturates (*n* = 2).

The results of our survey suggest potential gaps in both the quantity and quality of insomnia training in addiction psychiatry fellowship. Ensuring that all fellows receive insomnia training might be accomplished by adding the topic to the ACGME Milestones for Addiction Psychiatry Fellowship and the accompanying handbook for program directors. These milestones outline core competencies for addiction psychiatry fellows across six domains and describe target levels of performance at graduation [5]. Insomnia training in SUD might be most appropriately integrated into relevant domains by including knowledge of diagnosis and management under medical knowledge and appropriate clinical application under patient care. Improving the quality of insomnia treatment in SUD populations may need to be addressed more broadly than just in training. For example, the development and publication of evidence-based best practices for insomnia management for patients with SUDs could help to both standardize treatment strategies and reduce the use of potentially inappropriate treatments. Our literature search of PubMed and GoogleScholar found no such evidence-based guidelines.

The primary limitation of this study is the inability to calculate a response rate, which restricts assessment of potential response bias and may limit the generalizability of our findings. This concern can be best addressed through continued research on the insomnia training and management practices of addiction psychiatrists.

## Data Availability

De-identified data are available upon request to the corresponding author.
